# Left ventricular inflow propagation velocity for diastolic function testing: head-to-head comparison between velocity-encoded MRI and color M-mode Doppler echocardiography

**DOI:** 10.1186/1532-429X-15-S1-P54

**Published:** 2013-01-30

**Authors:** Pieter J van den Boogaard, Nina Ajmone Marsan, Jeroen J Bax, Albert de Roos, Jos J Westenberg

**Affiliations:** 1 Radiology, Leiden University Medical Center, Leiden, the Netherlands; 2 Cardiology, Leiden University Medical Center, Leiden, the Netherlands

## Background

The inflow propagation velocity (Vprop) of the early filling wave has been proposed as an accurate marker of left ventricular (LV) diastolic function [1]. Traditionally, Color M-mode echo Doppler is used for Vprop-assessment. However, this method has not been validated against an alternative modality such as velocity-encoded (VE) MRI for assessing Vprop. The purpose of this study was to compare Vprop assessed from high temporal VE MRI with Color M-mode echo Doppler in patients with ischemic cardiomyopathy.

## Methods

**Figure 1:**
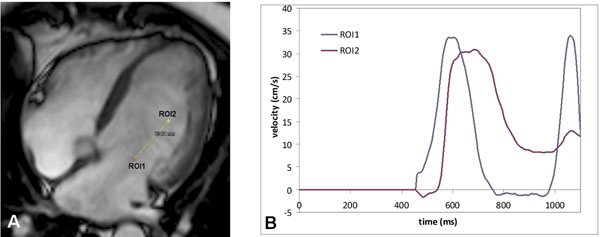
Example of Vprop-assessment with VE MRI. Regions-of-interest (ROIs) were positioned in a 4-chamber view at the mitral valve (ROI1) and at 37mm distal to the valve (ROI2), aligned along the direction of the inflow (A). Velocity was sampled at both ROIs and velocity-time curves were constructed (B). Inflow propagation was determined from the propagation of the Epeak.

In 36 patients (mean age 60±12 years; 25 men) with known ischemic cardiomyopathy and impaired LV systolic function, one-directional time-resolved VE MRI was performed on 1.5T MRI (Philips) to acquire the LV inflow pattern. A 4-chamber orientation was chosen with in-plane velocity-encoding in phase encoding (=long-axis) direction and velocity sensitivity 20cm/s. Effective temporal resolution of 6.5ms (true temporal resolution 2×TR=13ms) was achieved. The LV inflow pattern was sampled at the position of the mitral valve (MV) and at a location approximately 4cm distally, with regions-of-interest (ROIs) aligned along the visually-assessed inflow direction (Figure 1A). The inflow velocity-time curves were constructed from the mean velocities per cardiac phase sampled in each of these ROIs (size typically 2mm^2^) (Figure 1B). Phase unwrapping was used for aliasing correction. Early peak filling (Epeak) velocity was determined from the velocity sampled at the MV. The time-to-peak inflow velocity was determined from the velocity-time curves, and the distance between ROIs (Δx) and the difference in time-to-peak velocity (Δt) defined Vprop (=Δx/Δt).

For comparison purpose, patients underwent echocardiography within the same week of MRI to assess Vprop by Color M-mode echo Doppler as previously described [1]. Vprop from VE MRI was compared with echo Doppler, using paired t-test and Pearson correlation. When using a cut-off value for Vprop of 45cm/s to classify diastolic dysfunction [1], a cross-table was constructed to determine weighted kappa agreement between both modalities.

**Table 1:** Diastolic dysfunction classification using Vprop<45cm/s as cut-off criterion.

		Echo	
	
		≤45cm/s	>45cm/s
MRI	≤45cm/s	21	2
	>45cm/s	4	9

## Results

Assessment of Vprop from VE MRI showed good correlation with echo Doppler with Pearson R = 0.71 (p<0.001). A small statistically non-significant overestimation was present on VE MRI compared to echo Doppler of 3±24cm/s (p=0.40). Vprop was not statistically significant correlated with Epeak velocity (Pearsons R=0.14, p=0.41), both assessed with VE MRI.

VE MRI and echo Doppler showed good agreement (kappa 0.72), with sensitivity/specificity of 84%/82% for diastolic function classification by Vprop.

## Conclusions

VE MRI and Color M-mode echo Doppler showed good correlation and agreement for Vprop-assessment in ischemic cardiomyopathy regardless of LV inflow velocity, with a high sensitivity and specificity for VE MRI classifying diastolic dysfunction.

## Funding

STW project 11626
